# Plant hormone-mediated regulation of stress responses

**DOI:** 10.1186/s12870-016-0771-y

**Published:** 2016-04-14

**Authors:** Vivek Verma, Pratibha Ravindran, Prakash P. Kumar

**Affiliations:** Department of Biological Sciences, Faculty of Science, National University of Singapore, Singapore, 117543 Singapore; Present address: School of Biological and Biomedical Sciences, Durham University, South Road, Durham, DH1 3LE UK

**Keywords:** Abiotic stress, Biotic stress, Plant hormones, Crosstalk, Abscisic acid (ABA), Gibberellins (GA), Salicylic acid (SA), Jasmonates (JA)

## Abstract

**Background:**

Being sessile organisms, plants are often exposed to a wide array of abiotic and biotic stresses. Abiotic stress conditions include drought, heat, cold and salinity, whereas biotic stress arises mainly from bacteria, fungi, viruses, nematodes and insects. To adapt to such adverse situations, plants have evolved well-developed mechanisms that help to perceive the stress signal and enable optimal growth response. Phytohormones play critical roles in helping the plants to adapt to adverse environmental conditions. The elaborate hormone signaling networks and their ability to crosstalk make them ideal candidates for mediating defense responses.

**Results:**

Recent research findings have helped to clarify the elaborate signaling networks and the sophisticated crosstalk occurring among the different hormone signaling pathways. In this review, we summarize the roles of the major plant hormones in regulating abiotic and biotic stress responses with special focus on the significance of crosstalk between different hormones in generating a sophisticated and efficient stress response. We divided the discussion into the roles of ABA, salicylic acid, jasmonates and ethylene separately at the start of the review. Subsequently, we have discussed the crosstalk among them, followed by crosstalk with growth promoting hormones (gibberellins, auxins and cytokinins). These have been illustrated with examples drawn from selected abiotic and biotic stress responses. The discussion on seed dormancy and germination serves to illustrate the fine balance that can be enforced by the two key hormones ABA and GA in regulating plant responses to environmental signals.

**Conclusions:**

The intricate web of crosstalk among the often redundant multitudes of signaling intermediates is just beginning to be understood. Future research employing genome-scale systems biology approaches to solve problems of such magnitude will undoubtedly lead to a better understanding of plant development. Therefore, discovering additional crosstalk mechanisms among various hormones in coordinating growth under stress will be an important theme in the field of abiotic stress research. Such efforts will help to reveal important points of genetic control that can be useful to engineer stress tolerant crops.

## Background

The sensing of abiotic and biotic stresses initiates several complex signaling pathways in plants. Some of the early signaling events include alteration of intracellular Ca^2+^ concentration, production of secondary signaling molecules such as inositol phosphate and reactive oxygen species (ROS) as well as activation of kinase cascades. The increase in intracellular Ca^2+^ levels in response to the adverse environmental conditions is detected by calcium binding proteins that function as Ca^2+^ sensors [1]. The activated Ca^2+^ sensors can either bind to *cis*-elements in the promoters of major stress-responsive genes or can interact with DNA-binding proteins controlling these genes, thereby, resulting in their activation or suppression. Furthermore, elevated Ca^2+^ levels can activate calcium-dependent protein kinases (CDPKs), calcium/calmodulin-dependent protein kinases (CCaMKs) or phosphatases that in turn can phosphorylate/dephosphorylate specific transcription factors, thus, regulating expression levels of stress-responsive genes [2].

Ca^2+^ functions in concert with other important second messengers like ROS. A rapid increase in the rate of ROS production, known as ‘the oxidative burst’, occurs as a response to stress conditions [3]. The ROS molecules that mediate signaling functions include hydrogen peroxide (H_2_O_2_), singlet oxygen (^1^O_2_), hydroxyl radical and superoxide anion radical [4]. The activation of mitogen-activated protein kinase (MAPK) cascade by H_2_O_2_ and subsequent upregulation of specific stress-related genes in *Arabidopsis* is a perfect example of ROS-mediated stress-response [5]. Nonetheless, Ca^2+^ and ROS-mediated responses of plants to environmental constraints just form the tip of the iceberg. The mechanism of stress-response in plants is highly intricate and requires several integrated pathways to be activated in response to external stresses. Because of the complex interactions among various plant hormones and their ability to control a wide range of physiological processes, they serve as the key endogenous factors in mediating plant stress response. Moreover, with nine diverse groups of plant hormones participating in defense responses, their signaling pathways are intricately interconnected to facilitate the generation of a sophisticated and efficient stress response. Therefore, a concise overview of the role of plant hormones with special focus on how they crosstalk in regulating various stress responses will be provided in this review.

### Plant hormones as watchdogs of stress response

The major hormones produced by plants are auxins, gibberellins (GA), cytokinins (CK), abscisic acid (ABA), ethylene (ET), salicylic acid (SA), jasmonates (JA), brassinosteroids (BR) and strigolactones. Among these, ABA, SA, JA and ET are known to play major roles in mediating plant defense response against pathogens and abiotic stresses [6, 7]. Typically, ABA is responsible for plant defense against abiotic stresses because environmental conditions such as drought, salinity, cold, heat stress and wounding are known to trigger increase in ABA levels [8, 9]. Contrastingly, SA, JA and ET play major roles in response to biotic stress conditions as their levels increase with pathogen infection [6]. However, the mechanism of stress-response is not solely restricted to these hormones. Recent studies have provided substantial evidence for the crosstalk of ABA, SA, JA and ET with auxins, GAs and CKs in regulating plant defense response [6, 10, 11]. Hence, the regulatory roles of ABA, SA, JA and ET and their crosstalk with other hormones will be discussed here.

#### Role of ABA in plant defense response

The prominent contribution of ABA to plant defense response against abiotic stress conditions has long been studied. Under osmotic conditions such as high salinity and drought, ABA is known to stimulate short-term responses like closure of stomata, resulting in maintenance of water balance [12] and longer term growth responses through regulation of stress-responsive genes. ABA accumulates upon occurrence of osmotic stresses because expression levels of several ABA biosynthesis genes, such as *ZEAXANTHIN EPOXIDASE* gene (*ZEP*; also known as *LOS6* [for *LOW EXPRESSION OF OSMOTIC STRESS-RESPONSIVE* gene 6]/*ABA1*), the *ALDEHYDE OXIDASE* gene (*AAO3*), a *9-CIS-EPOXYCAROTENOID DIOXYGENASE* gene (*NCED3*), and the *MOLYBDENUM COFACTOR SULFURASE* gene (*MCSU*; also known as *LOS5/ABA3*), are upregulated by drought and salt stress [13].

Furthermore, promoter analysis of ABA-responsive genes has shown the presence of multiple *cis*-elements, designated as ABA-responsive elements (ABREs; PyACGTGG/TC), in their promoters [14, 15]. The basic leucine zipper transcription factors, ABRE-BINDING PROTEINS (AREBs)/ABRE-BINDING FACTORS (ABFs) can bind to ABRE and result in the upregulation of ABA-responsive genes [16]. The ABA-mediated phosphorylation of ABFs is necessary for their activation [17]. The induction of AREB1/ABF2, AREB2/ABF4 and ABF3 by dehydration, high salinity and ABA treatment and enhanced drought tolerance by plants overexpressing these factors further validates the significance of these proteins and hence ABA in abiotic stress response [16].

Other transcription factors from the MYC, MYB and NAC protein families are also known to function in an ABA-dependent manner [18, 19]. Overexpression of AtMYC2 and AtMYB2 transcription factors, besides exhibiting an ABA-hypersensitive response, also improved osmotic stress tolerance of transgenic plants [19, 20]. Likewise, transgenic plants overexpressing RD26 (a stress-inducible NAC transcription factor) showed high sensitivity to ABA and thus an upregulation of ABA- and stress-responsive genes [18].

Interestingly, studies in the recent past have highlighted that ABA-dependent pathways also play an important role in the regulation of dehydration-responsive element (DRE)-BINDING PROTEIN (DREB) transcription factors, under osmotic stress conditions [8]. Exhibition of ABA-hypersensitive response by transgenic plants overexpressing DREB2C and interaction of DREB1A and DREB2A with ABF2 and that of DREB2C with ABF3 and ABF4 confirmed the involvement of ABA in regulation of DREB transcription factors [21]. This was further validated by yeast one-hybrid and chromatin immunoprecipitation (ChIP) assays indicating the binding of AREB1, AREB2 and ABF3 to the DREB2A promoter, resulting in the activation of DREB2A in an ABA-dependent manner [22]. Thus, it is evident that ABA employs a sophisticated process for mediating plant defense responses against abiotic stresses.

#### Role of SA, JA and ET in plant defense response

SA, JA and ET are mainly known to play significant roles in regulating plant defense responses against various pathogens and pests [6]. SA is generally involved in the activation of defense response against biotrophic and hemi-biotrophic pathogens [23], whereas, JA and ET are responsible for defense against necrotrophic pathogens and herbivorous insects [24, 25].

SA synthesis takes place in response to detection of phytopathogens. Once SA pathway is activated at the site of infection, a defense response is often triggered in distal plant parts to protect undamaged tissues. This long-lasting and broad-spectrum induced resistance is referred to as systemic acquired resistance (SAR). Mutants insensitive to SA or defective in SA accumulation exhibit enhanced susceptibility to pathogens. Moreover, increase in SA levels in pathogen-exposed tissues results in the induction of PATHOGENESIS RELATED (PR) genes. These PR genes are a diverse group that encode several proteins with antimicrobial activity and hence increase resistance to a wide range of pathogens [26]. One of the key regulatory elements in SA-dependent activation of PR genes is NON-EXPRESSOR OF PR GENE 1 (NPR1). It is known that SA regulates the deoligomerization of NPR1 into its active monomeric forms. The monomers localize into the nucleus and interact with TGA class of bZIP transcription factors, which in turn, facilitate PR gene expression and subsequent defense response [23]. However, recent studies have highlighted that NPR1 is a SA receptor and SA directly regulates the conformation of NPR1 by deoligomerizing NPR1 into a dimer [27]. Several WRKY transcription factors are also known to play important roles, downstream of NPR1, in mediating the defense responses in plants [28, 29].

An increase in JA levels in response to pathogen infection clearly highlights its involvement in plant defense response. Besides, JA signaling also plays a prominent role in defending plants against many herbivores, such as, caterpillars, spider mites, beetles, thrips and mirid bugs [24]. JA-responsive gene expression for defense response is mainly mediated by a transcription factor JASMONATE INSENSITIVE 1/MYC2 (JIN1/MYC2) [28]. Several members of the APETALA2/ETHYLENE-RESPONSIVE FACTOR (AP2/ERF) family have also been reported to participate in JA-regulated stress responses [31, 32]. ERF1, ERF2, ERF5 and ERF6 control the expression levels of JA-responsive marker gene *PLANT DEFENSIN 1.2* (*PDF1.2*) and provide resistance against necrotrophic pathogens [33–35]. Further, a repressor protein, JASMONATE-JIM-DOMIN (JAZ), also plays a crucial role in JA response under stress conditions [36]. In the absence of JA-Ile, the bioactive JA [37], JAZ proteins interact with JIN1/MYC2 and inhibit transcriptional regulation of JA-responsive genes. In JA-stimulated conditions, JA-Ile binds to its receptor, an F-box protein CORONATINE INSENSITIVE1 (COI1), and leads to 26S proteasome-mediated degradation of JAZ, thereby allowing MYC2 to upregulate the expression level of JA target genes [36]. Recent studies show that MYC2 is post-translationally modified by phosphorylation at Thr328 residue to stimulate its transcription activity [38]. However, the modified MYC2 is unstable and degraded by Plant U-box protein (PUB10) which functions as an E3 ligase [39]. This facilitates turnover of MYC2, thereby facilitating dynamism and fine-tuning of JA responses by MYC2.

ET plays diverse roles in plant defense response [6, 25]. ERFs are the major downstream regulatory factors of ET signaling pathway in stress-responses. The transcription factor ETHYLENE INSENSITIVE3 (EIN3) was suggested to induce *ERF1* gene expression in response to ET and activate defense responses [40]. Another positive regulator of ET signaling is EIN2. In the absence of ET, CONSTITUTIVE TRIPLE RESPONSE (CTR1) represses EIN2. Upon perception of ET by its receptor ETHYLENE RESPONSE 1 (ETR1), the repression on EIN2 is relieved, thereby activating ET signaling [41]. ET can crosstalk with SA and JA pathways either antagonistically or by promoting them to achieve tailored defense responses.

From the discussion so far, it is clear that plants are challenged by a variety of stress conditions during their life cycles. Consequently, plants have evolved a multitude of stress responses, where response to a particular stress condition is primarily under the control of a specific plant hormone. Nevertheless, recent findings have proven beyond doubt that besides playing critical roles at individual levels, different plant hormones also crosstalk to facilitate the coordination of an array of genes and their regulators involved in stress remediation [42]. Therefore, for gaining a better insight into the plant defense mechanism, it is imperative to understand the intricate nexus of crosstalk among different plant hormones.

#### Hormonal crosstalk in plant defense

The signaling pathways of ABA, SA, JA and ET are known to interact among themselves at various nodes, such as hormone-responsive transcription factors to regulate plant defense response. However, it is noteworthy that whole plant adaptation and sustained growth are the key features of a proper defense response under stress conditions. Therefore, the crosstalk of ABA, SA, JA and ET with the major growth promoting hormones, i.e. auxins, GAs and CKs plays an important role in mediating the stress response. Also, the defense responses activated in plants in response to different stresses depends on the type of crosstalk (positive or negative) between the hormone signaling pathways rather than solely on the individual contributions of each hormone. Hence, this section will provide a brief overview of the crosstalk among different plant hormones and the regulatory role of this crosstalk in plant defense response. Subsequently, the crosstalk of GA with ABA, mediated by DELLAs, in regulating the balance between seed dormancy and germination, a key mechanism for evading early abiotic stress conditions, will be discussed in more detail to provide a deeper insight into the complexities of hormonal crosstalk involved in mediating stress responses.

The signaling pathways of SA and JA are known to intersect at various points because SA and JA regulate biotic stress responses antagonistically [6]. This antagonistic relation was first reported in tomato, where JA-related wound response was inhibited by aspirin, an acetylsalicylic acid drug [43]. Studies have shown NPR1 to be a key player in the antagonistic crosstalk of SA and JA. The SA-facilitated suppression of JA-responsive genes like *LIPOXYGENASE 2* (*LOX2)*, *VEGETATIVE STORAGE PROTEIN (VSP)*, and *PDF1.2* was abolished in *npr1* mutant plants [44].

**Fig. 1:**
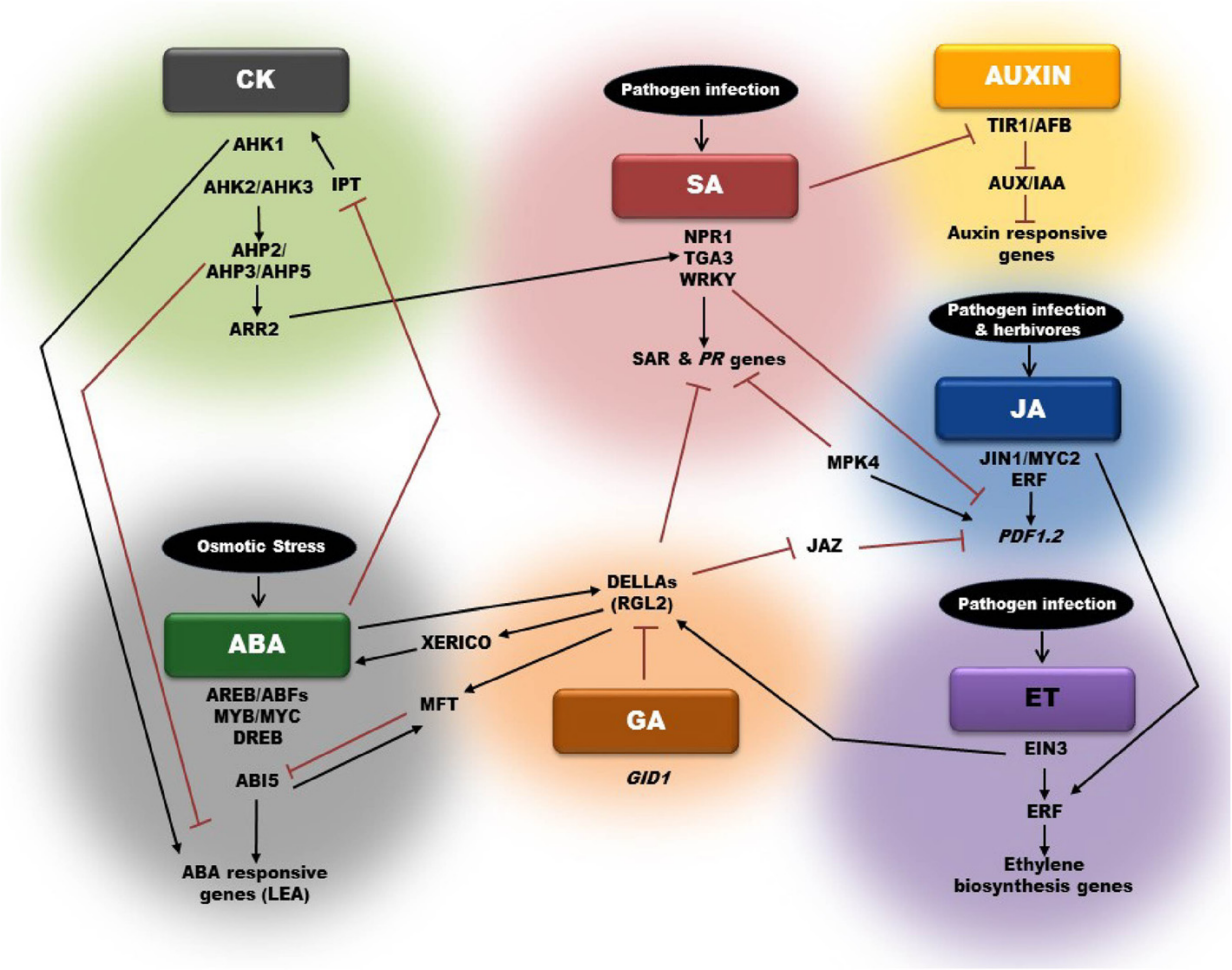
An overview of plant hormone signaling networks and their crosstalk in stress responses. ABA, SA, JA and ET are major players in stress response, with ABA mainly regulating osmotic stresses. SA, JA and ET control biotic stress responses. ABA and GA signaling pathways interact, with DELLAs serving as a crosstalk point, to influence the balance between seed dormancy and germination. SA and JA pathways are antagonistically regulated by several transcription factors. JA-ET crosstalk synergistically. Auxins, GAs and CKs participate in biotic stress responses via SA signaling pathway. CKs also crosstalk with ABA and function in drought and salinity stress responses. Arrows represent positive regulation (accumulation of transcripts, proteins or hormones), and blocked arrows represent negative regulation. For abbreviations refer to text

The WRKY 70 transcription factor is also a key component mediating the antagonistic interaction between the two hormones. Overexpression of WRKY70, on the one hand, resulted in constitutive expression of SA-responsive *PR* genes, and on the other hand, caused repression of JA-responsive *PDF1.2* gene [45]. Likewise, *mpk4 (MAP kinase 4)* knock-out mutants in *Arabidopsis* that exhibited constitutive SAR had higher expression levels of *PR* genes, but the expression levels of JA-responsive genes (*PDF1.2* and *THI2.1)* were impaired [46] (Fig. 1). Although most studies prove antagonistic interaction between SA and JA, synergistic interactions have been observed at low SA-JA concentrations and by simultaneous induction of both defenses [45, 46].

In contrast to the largely antagonistic functions of SA and JA, JA and ET operate synergistically in regulating defense related genes after pathogen infection. Both JA and ET pathways induce/stabilize EIN3 and thus exhibit synergy in root hair development and resistance to necrotrophs [49]. A positive JA-ET interaction causes induction of genes encoding for proteinase inhibitors in response to wounding in tomato [50]. Likewise, both JA and ET are required to simultaneously activate expression of *ERF1*, and thereby activate *PR* genes [32]. Recent studies in *Arabidopsis* showed that JA and ET signaling pathways could also behave antagonistically against attack by insects and herbivores. JA-activated MYC2 was found to interact with ET-stabilized EIN3 and repress its downstream functions. Conversely, EIN3 represses MYC2 and thereby inhibits JA-regulated defense response against herbivores [51]. Besides, ET is also known to crosstalk with ABA for abiotic stress responses because DREBs belong to the ERF family of transcription factors that are induced by ethylene Moreover, ET also counteracts ABA action in seeds and thereby improves dormancy release and germination [52].

Auxins have long been known to be responsible for regulating plant development. However, several recent studies have also highlighted their roles in stress response. Auxins associate with ethylene to regulate root development and architecture, which is a key aspect of drought and salinity tolerance [53]. The negative regulation of lateral root formation and positive regulation of adventitious root formation by ethylene via modulation of auxin transport provides another instance of auxin-ethylene crosstalk in modifying root architecture [54]. Furthermore, treatment of *Arabidopsis* plants with benzothiadiazole S-methyl ester (BTH), an SA analog, resulted in the suppression of several auxin-responsive genes. SA signaling represses the expression of the *TRANSPORT INHIBITOR RESISTANT 1 (TIR1)/ AUXIN SIGNALING F-BOX (AFB)* genes, resulting in stabilization of auxin repressor protein AUX/IAA and thus repression of auxin responses [55] (Fig. 1). A majority of the auxin responsive genes were also suppressed after induction of SAR, clearly suggesting that auxin promotes disease susceptibility, and enhanced resistance to diseases would necessitate repression of auxin signaling. Taken together, auxin acts as a key constituent of the signaling network of hormones mediating the regulation of defense response.

The role of CKs in biotic stress response has been demonstrated by several studies [53, 56, 57]. Transgenic *Arabidopsis* plants having stabilized CK levels exhibited enhanced resistance against infection with hemi-biotrophic pathogen *Verticillium longisporum* [56]. Cytokinins are also known to crosstalk with SA signaling cascade to regulate plant defenses. For instance, cytokinin-activated transcription factor ARABIDOPSIS RESPONSE REGULATOR 2 (ARR2), a type B ARR, interacts with a bZIP-type transcription factor TGA3 and promotes SA defense responses in an NPR1-dependent manner [57]. Similarly, the synergistic interaction between SA and CK, in an OsNPR1- and WRKY45-dependent manner, has been shown to increase rice resistance to the blast fungus *Magnaporthe oryzae* [58].

Analyses of gene expression studies revealed that exogenous ABA application resulted in suppression of *ISOPENTENYL TRANSFERASE*, a cytokinin biosynthesis gene [59]. A majority of the *CYTOKININ OXIDASES* were also suppressed when treated with ABA. Likewise, gain- and loss-of-function studies of ARABIDOPSIS HISTIDINE KINASEs (AHKs), which function as cytokinin receptors, indicated that AHK1 acts as a positive regulator of drought and salinity response and also ABA signaling, while AHK2 and AHK3 negatively regulate osmotic stress response and ABA signaling [60]. Also, ARABIDOPSIS HISTIDINE PHOSPHOTRANSFER PROTEINs (AHPs), namely AHP2, AHP3, and AHP5, negatively control responses to drought stress because the loss-of-function of these three *AHP* genes resulted in up-regulation of ABA-responsive genes and thus a strong drought tolerant phenotype [11] (Fig. 1).

#### DELLAs modulate early defense by mediating GA-ABA crosstalk in seeds

Seed dormancy is an adaptive trait that delays germination until ambient conditions are favorable for survival. It protects seeds from harsh environmental conditions (abiotic stress) under which the probability of survival for seedlings is very low. Thus, dormancy is the first and foremost defense response in the seed stage of plants. Dormancy is maintained by ABA whose levels rise during embryogenesis and are high in mature seeds [61]. It has been suggested that ABA inhibits water uptake by preventing cell wall loosening of the embryo and thereby reduces embryo growth potential [62]. ABA also causes the accumulation of ABSCISIC ACID INSENSITIVE 5 (ABI5), a basic leucine zipper transcription factor that causes growth arrest by recruitment of some of the *LATE EMBRYOGENESIS ABUNDANT* (*LEA*) genes, whose products confer osmotolerance to the embryo under harsh environmental conditions [63, 64].

The repressive effects of ABA are overcome by GAs, a class of phytohormones that has long held a prominent role in plant growth and development. Gibberellins promote germination of mature seeds when favorable conditions of light, temperature and moisture set in. Germination begins with water uptake by seeds and terminates with the emergence of the radicle [65]. GA biosynthesis and response pathways are activated during seed imbibition resulting in an increase in bioactive GAs. These GAs induce genes encoding for enzymes such as ENDO-β-1,3 GLUCANASE [66], β -1,4 MANNAN ENDOHYDROLASE [65, 67] that hydrolyze the endosperm and release the inhibitory effects of ABA on embryo growth potential [68]. This means that ABA and GA have an antagonistic relationship; favorable environmental conditions lead to high GA and low ABA levels in seeds whereas unfavorable conditions cause the reverse ratio. Thus, GA-ABA crosstalk regulates the balance between seed dormancy and germination, a key mechanism for evading early abiotic stress conditions.

Several GA signaling components have been identified by genetic studies [69, 70]. Positive regulators of GA signaling, mutants of which typically exhibit a dwarfed phenotype with dark green compact leaves, delayed flowering, reduced fertility, and no or poor seed germination have been identified. Some of them include the rice *dwarf1* (*d1*) [71] and *GA-insensitive dwarf2* (*gid2*) [72] as well as the *Arabidopsis sleepy1* (*sly1*) [73] mutation. GA signaling is also known to be negatively regulated by a class of repressors called DELLA proteins that belong to the GRAS family of transcription factors. These DELLA proteins are known to function as integrators of the GA and ABA triggered signaling pathways [74]. They are named after their highly conserved N-terminal DELLA motif, which mediates GA-responsiveness [75, 76]. A single DELLA protein is present in rice and barley (SLENDER RICE1 [SLR1] and SLENDER1 [SLN1], respectively) and it functions to repress every aspect of GA responses in these species [77]. Surprisingly, five DELLA proteins have been identified in *Arabidopsis*: GA INSENSITIVE (GAI), REPRESSOR OF GA1-3 (RGA), RGA-LIKE1 (RGL1), RGL2 and RGL3 [75, 78–80]. Under stress conditions, this growth-restraining function of DELLAs helps to improve survival by diverting limited resources to defense responses. RGA and GAI are the major repressors of stem elongation [78, 81], RGA, RGL1 and RGL2 impair flower development [82–84] whereas RGL2 is the major repressor of seed germination and its function is enhanced by GAI, RGA and RGL1.

A single knockout of *RGL2* is able to rescue the germination defect of the GA biosynthetic mutant *ga1-3* even in the absence of exogenous GA, thereby mimicking WT germination. Thus, RGL2 has been proposed as the main DELLA protein that needs to be inactivated during GA-induced breaking of dormancy [85, 86]. Perception of GA signal leads to the destruction of DELLAs via the 26S proteasome pathway and thus promotes seed germination [87, 88].

In addition to GA, studies have shown the involvement of RGL2 in ABA signaling as well. RGL2 was shown to stimulate *XERICO* expression, which encodes a RING-H2 factor, to elevate endogenous ABA levels and thus ABI5 activity, especially under low-GA conditions [89, 90]. ABA, in turn, enhances the *RGL2* expression [91]. Recently, it has been shown that high ABA levels in imbibed dormant seeds requires the permanent expression of RGL2 [92]. In contrast, non-dormant seeds expressed RGL2 only transiently upon imbibition and thereby germinated. Therefore, it is likely that RGL2 may integrate GA and ABA signaling pathways in regulating seed dormancy. Accordingly, it has been found that RGL2 upregulates MOTHER OF FT AND TFL1 (MFT), which encodes a phosphatidylethanolamine-binding protein, by binding to its promoter region through an unknown complex [93]. *MFT* expression is also directly regulated by ABA-INSENSITIVE3 (ABI3) and ABI5, with the former acting as a repressor and the latter as a promoter. MFT, in turn, directly represses *ABI5,* thereby providing a negative feedback regulation of ABA signaling. Thus, MFT serves as a convergence point of ABA and GA signaling pathways downstream of RGL2 during seed germination [93] (Fig. 1). SPATULA (SPT) transcription factor has been shown to drive both “dormancy-repressing” and “dormancy-promoting” routes by regulating the expression of ABI4, ABI5, RGA, RGL3 and MFT [94]. Hence, GA and ABA are the key plant hormones that regulate the fine balance between seed dormancy and germination and thus provide the first level of defense.

Crosstalk between GA and JA pathways also occurs via DELLA proteins. Studies have shown that the DELLAs can interact with JAZ1, the key repressors of JA signaling, thus preventing JAZ1-mediated repression of transcription [95]. For instance, JA signaling induces expression of *RGL3*, which competes with MYC2 for binding to JAZ1 and JAZ8 [96]. Thereby, RGL3 positively regulates JA-mediated resistance to necrotrophs and hemi-biotrophs. By interfering with GA-mediated degradation of DELLA proteins, JA prioritizes defensive over growth-related pathways [97, 98]. Also, another significant crosstalk is illustrated by delayed induction of the JA/ET dependent gene marker *PDF1*.2 in the *Arabidopsis* quadruple-DELLA mutant lacking GAI, RGA, RGL1, and RGL2 proteins, thereby making them more susceptible to necrotrophs [10]. Because SA works antagonistic to JA/ET, the SA-dependent *PR1* and *PR2* transcripts were highly induced in infected quadruple-DELLA mutant providing them resistance to hemi-biotrophs [10].

GAs also crosstalk with several other hormones to regulate plant growth and development in response to stresses. DELLAs have been shown to integrate ET signaling in promoting salt tolerance [99]. The root growth in quadruple-DELLA mutant seedlings was less inhibited by salt than that of the wild type, thereby suggesting that salt slows growth by means of a DELLA-dependent mechanism. Salt-activated ET signaling was found to confer salt tolerance by enhancing the function of DELLAs. Crosstalk with DELLAs via the CTR1-dependent ET response pathway occurs downstream of EIN3 [99]. Similarly, the cold-induced *CBF1/DREB1b*, member of the AP2/ETHYLENE-RESPONSIVE ELEMENT BINDING PROTEIN(EREB), confers freezing tolerance and slows growth by allowing the accumulation of DELLAs [100]. Thus, GAs function in both salt and cold stress response pathways via the DELLA proteins and show significant crosstalk with ET signaling.

## Conclusions

From the foregoing discussion it is clear that plants utilize elaborate signaling pathways in responding to stresses. In addition to other small molecules such as Ca^2+^ and ROS, plant hormones trigger specific signal cascades upon abiotic or biotic stress perception. The fluctuations in several key hormone levels such as ABA, ET, SA and JA occur as early responses to stress. These affect metabolic processes that ultimately result in an altered growth pattern suitable for withstanding the environmental stress. Recent research findings have helped to clarify the elaborate signaling networks and the sophisticated crosstalk occurring among the different hormone signaling pathways. Such crosstalk helps to integrate various stress signal inputs and allows plants to respond to them appropriately. The readjustment of growth responses and acquisition of enhanced levels of tolerance to the stresses are key to the survival of plants. At the molecular level, these are facilitated by the presence of multiple signal intermediates for each hormone and their ability to crosstalk at various signaling levels. These have been illustrated in the present review with examples drawn from selected abiotic and biotic stress responses. The discussion on seed dormancy and germination serves to illustrate the fine balance that can be enforced by the two key hormones ABA and GA in regulating plant responses to environmental signals.

It is apparent that the signaling interactions among multiple phytohormones are rather common in controlling various growth and developmental processes. Plants may control hormone action at various points, e.g., by regulating the biosynthesis of a given phytohormone, by modifying the available pool of hormone molecules or by elaborate regulation of the signaling process. Plant biologists have long recognized the conundrum of extreme pleiotropy in phytohormone action, namely, the regulation of multiple developmental events by a given phytohormone. The more recent discoveries of the presence of multiple receptors and signaling intermediates (e.g., over 20 response regulators in cytokinin signaling, over 20 AUX/IAA genes in auxin signaling or the presence of a similar number of JA signaling intermediates) shows the molecular players behind the extensive pleiotropy in phytohormone action. The intricate web of crosstalk among the often redundant multitudes of signaling intermediates is beginning to be better understood. Future research employing genome-scale systems biology approaches to solve problems of such magnitude will undoubtedly lead to detailed understanding of plant development. Therefore, revealing additional crosstalk mechanisms among various hormones in coordinating growth under stress will be an important theme in the field of abiotic stress research. Furthermore, such a paradigm of phytohormone signal crosstalk will present valuable new avenues for genetic improvement of crop plants needed to meet the future food production targets in the face of global climate change. Thus, manipulation of phytohormone action at the right developmental stages and appropriate tissues/organs will be an attractive avenue to understand and engineer stress tolerance.

### Availability of data and materials

All the data supporting our review is contained within the manuscript.

## Abbreviations

ABA: abscisic acid
ABF: ABRE-BINDING FACTOR
ABI5: ABSCISIC ACID INSENSITIVE 5
AHKs: ARABIDOPSIS HISTIDINE KINASEs
AHPs: ARABIDOPSIS HISTIDINE PHOSPHOTRANSFER PROTEINs
AP2/ERF: APETALA2/ETHYLENE-RESPONSIVE FACTOR
AREB: ABRE-BINDING PROTEIN
ARRs: ARABIDOPSIS RESPONSE REGULATORs
BR: brassinosteroid
CK: cytokinin
CTR1: CONSTITUTIVE TRIPLE RESPONSE
DREB: (DRE)-BINDING PROTEIN
EIN2: ETHYLENE INSENSITIVE2
EIN3: ETHYLENE INSENSITIVE3
ET: ethylene
GA: Gibberellin
GAI: GA INSENSITIVE
H_2_O_2_: hydrogen peroxide
JA: jasmonic acid
JAZ: JASMONATE-JIM-DOMIN
JIN1/MYC2: JASMONATE INSENSITIVE 1/MYC2
MFT: MOTHER OF FT AND TFL1
MPK4: MAP KINASE 4
NPR1: NON-EXPRESSOR OF PR GENE 1
PDF1.2: PLANT DEFENSIN 1.2
PR: PATHOGENESIS RELATED
RGA: REPRESSOR OF GA1-3
RGL1: RGA-LIKE1
RGL2: RGA-LIKE2
RGL3: RGA-LIKE3
ROS: reactive oxygen species
SA: salicylic acid
SAR: systemic acquired resistance
TIR1/AFB: TRANSPORT INHIBITOR RESISTANT 1/ AUXIN SIGNALING F-BOX
